# Unusual Manifestation of Extrapulmonary Tuberculosis

**DOI:** 10.1155/2013/353798

**Published:** 2013-04-28

**Authors:** Aisha A. Alghamdi, Faiza S. Awan, Iqbal H. Maniyar, Naif A. Alghamdi

**Affiliations:** Medical Department, King Abdulaziz University Hospital (KAUH), P.O. Box 80200, Jeddah 21589, Saudi Arabia

## Abstract

Though commonly encountered, extrapulmonary tuberculosis (TB) can sometimes present with variable clinical picture intricating the diagnosis (Avasthi et al., 2010). The nonspecific symptoms include pyrexia of unknown origin, hepatosplenomegaly, lymphadenopathy, meningitis, and, rarely, variety of hematological abnormalities, namely, anemia, pancytopenia, and leukemoid reaction (Avasthi et al., 2010). When it presents with bone marrow (BM) involvement, prognosis is usually poor (Avasthi et al., 2010, Qasim et al., 2003, and Singh et al., 2001). We, hereby, report a case of extra-pulmonary TB with a six-month history of fever associated with dizziness, fatigability, and cough. During the hospital stay, the patient showed a spectrum of interesting hematological findings, including severe pancytopenia on peripheral smear, necrotizing caseating granulomas consistent with TB on bone marrow examination. The patient showed a good clinical as well as hematological response to antituberculosis treatment. This paper highlights the significance of a hematological picture in the final confirmation of TB, which may otherwise be passed off as nutritional or other unrelated causes.

## 1. Introduction

Tuberculosis (TB) is a contagious infection that can present with a variable clinical picture, hence, making the diagnosis difficult [1]. Hematological abnormalities associated with extrapulmonary TB include anemia of different types, leukemoid reaction, and rarely pancytopenia [1]. Bone marrow biopsy has been widely used as one of the diagnostic tools when blood counts show a picture of pancytopenia [1–3]. Though considered a treatable condition, bone marrow tuberculosis has been reportedly associated with fatal outcome [2–5].

We describe the case of an immunocompetent patient who presented with fever of unknown origin and hematological derangements. The purpose of this paper is to draw attention to the importance of correlating the uncommon presentation of a commonly encountered condition and highlighting the fact that the prognosis of bone marrow TB depends largely on thorough intervention, timely diagnosis, and rapid initiation of treatment.

## 2. Case Report

A 50-year-old Eritrean male, a resident of the same city, was admitted with a six-month history of on and off moderate to high grade fever ranging from 38 to 39°C. It was associated with productive cough, weight loss of 8 to 10 kilograms, dizziness, and generalized fatigability. There was no significant past medical history. On examination, the patient had high grade fever (39°C) with relative bradycardia and heart rate around 74 beats per minute. He had mild hepatosplenomegaly but no superficial lymphadenopathy, and there were no signs of meningeal irritation. A provisional diagnosis was made for TB. Differential diagnoses included leishmaniasis, brucellosis, hepatitis B and C, AIDS, hematologic and solid tumor malignancy, and connective tissue disease. Further investigations were asked to confirm the diagnosis.

**Figure 1:**
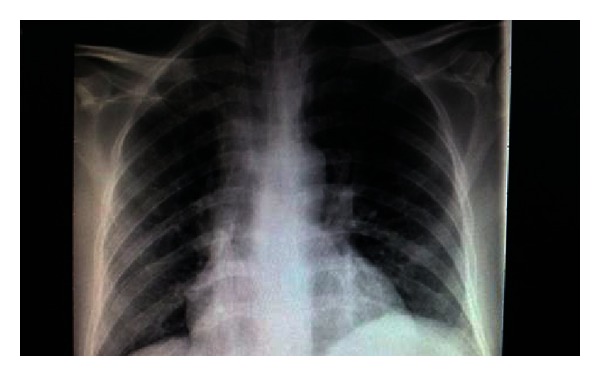
Chest X-ray.

Chest X-ray revealed a slightly widened anterior mediastinum (Figure 1). A complete blood count revealed severe anemia with hemoglobin of 2.3 g/dL (reference range 14.0–17.5), leucopenia 337/*μ*L (reference range 1000–4800), and normal platelet count of 297 × 10^3^/*μ*L (reference range 150–450). Liver function tests (LFTs), blood urea, and electrolytes were within normal range. Malaria film, autoimmune profile, and peripheral blood film were unremarkable. Lactate dehydrogenase was 902 U/L (reference range 100–190), erythrocyte sedimentation rate was 72 mm/h (reference range 1–20), and C-reactive protein was 132 mg/L (reference range 0–3). Lipid profile showed hypertriglyceridemia, with triglycerides around 2.94 mmol/L (reference range 0.30–2.30). Ferritin level was 19,948 ng/ml (reference range 30–400). Mantoux test was negative, and repeated sputum and blood cultures showed no growth. Hepatitis profile, human immunodeficiency virus (HIV) serology, and fecal occult blood were negative. Parasite serology showed high titre of schistosomiasis. Adult echocardiography showed no evidence of pericardial effusion or vegetations. Chest computed tomography (CT) scan showed multiple subcentimeter lymph nodes inaccessible to biopsy. An abdominal CT scan showed multiple sites of splenic infarction with no abdominal and pelvic lymphadenopathies.

**Figure 2:**
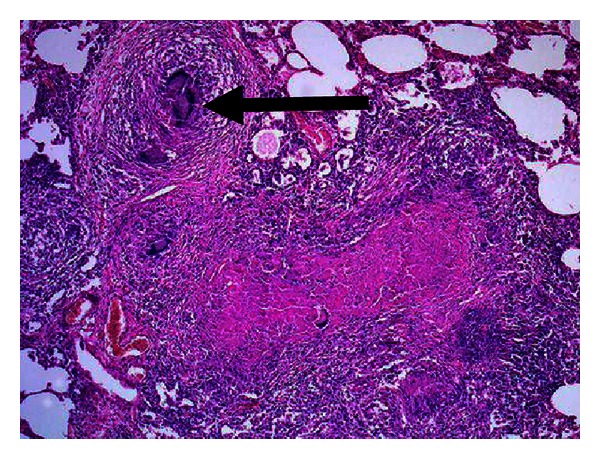
Arrow indicating caseating granuloma.

During hospitalization, the patient developed pancytopenia with hemoglobin of 2.0 g/dL (reference range 14.0–17.5), leucopenia 266/*μ*L (reference range 1000–4800), and platelet count of 23 × 10^3^/*μ*L (reference range 150–450), as well as hepatic derangement with direct bilirubin around 12.6 umol/L, alanine aminotransferase (ALAT) 256 IU/L, aspartate aminotransferase (ASAT) 277 IU/L, and alkaline phosphatase (ALP) 440 IU/L evident on repeated blood counts and liver function tests, respectively. Bone marrow biopsy showed hypocellular marrow infiltrated with caseating granuloma consistent with tuberculosis. In addition, acid fast bacilli were identified by Ziehl-Neelsen (ZN) stain (Figure 2). The patient was started on modified antituberculosis treatment including ethambutol, amikacin, and moxifloxacillin. Isoniazid and rifampicin were not included initially because the patient's liver profile was abnormal. These were added two weeks after initiation of anti-TB treatment and administered at half the usual dose, followed by full doses subsequent to improvement in LFTs about one month after diagnosis. The patient showed a good response within two weeks after initiation of treatment. Blood counts and liver enzymes improved gradually. The patient also received praziquantel 20 mg/kg body weight three times as a one-day treatment for schistosomiasis. Bone marrow biopsy was repeated to rule out any other underlying pathology. He showed complete recovery after six months of anti-TB treatment.

## 3. Discussion

Tuberculosis is one of the oldest and most commonly encountered diseases [1]. Although there is a significant steady decline in the incidence of active pulmonary tuberculosis due to early diagnosis and prompt treatment, the incidence of extrapulmonary TB has remained constant particularly due to a delay in recognizing the condition when the presenting clinical scenario consists mostly of nonspecific extrapulmonary symptoms [1, 4].

Extrapulmonary TB is considered a treatable disease with good outcome, requiring strict compliance [4, 6, 7]. When it presents with bone marrow involvement, the outcome depends largely on timely diagnosis and early initiation of treatment [5, 6].

The baseline workup towards the definite diagnosis of TB is usually a noninvasive approach and has significant yield [6]. Imaging studies did not prove very helpful in our case as his chest X-ray did not show the characteristic military pattern, and subcentimeter lymph nodes seen on chest CT were inaccessible for biopsy. The tuberculin skin test was negative, but skin tests are unreliable as these are usually negative in patients with extrapulmonary TB [8].

Extrapulmonary TB can present with variable hematologic abnormalities including anemia, leucopenia, leukocytosis, thrombocytopenia, thrombocytosis and monocytosis, and rarely pancytopenia [1, 2, 8]. In our case, laboratory investigations revealed hematological and biochemical abnormalities that included pancytopenia and raised ferritin level about 50 times above the normal upper limit. Several factors are considered to cause pancytopenia in disseminated or extrapulmonary tuberculosis including hypersplenism [1], histiocytic hyperplasia [1], maturational arrest [1], or infiltration of the bone marrow by caseating or noncaseating granulomas causing reversible or irreversible fibrosis [1, 7]. In the literature, there is no systematic pattern of diagnostic approach, and several diagnostic tests including invasive procedures have been used to confirm the diagnosis [6]. In our case, examination of the bone marrow was requested when the patient developed pancytopenia. The findings were consistent with bone marrow tuberculosis.

The incidence of bone marrow granuloma ranges from 0.38% to 2.2% [3, 5]. In contrast to good prognosis of pulmonary TB, the literature review of various similar reported cases of bone marrow TB has revealed high mortality in the range of fifty to almost hundred percent. Certain factors are thought to contribute to the variable outcome such as disease severity, other underlying pathologies leading to immunocompromised state, immunosuppressive therapies, and delay in initiation of appropriate treatment [1, 9]. Another contributing factor to poor outcome is macrophage-activating syndrome (MAS), which is a nonspecific clinical syndrome comprising of pancytopenia, hypertriglyceridemia, and hyperferritinemia [2] as was the case in our patient. A retrospective chart review of bone marrow TB was done during the period from 1990 to 2002 at King Faisal Specialist Hospital and Research Center, Riyadh, which showed a mortality rate of 50% [10]. The high mortality in that study was attributed to the delay in presentation [10].

Although certain poor prognostic factors such as MAS, chronicity, and caseating tubercular granulomas in the bone marrow were present, our patient showed good subjective response with subsidence of symptoms within few weeks after following the initiation of the treatment. The favorable outcome in our patient is thought to be due to an early diagnosis, rapid start of treatment, good compliance to anti-TB medication, and thorough followup.
